# Conformational Response of 30S-bound IF3 to A-Site Binders Streptomycin and Kanamycin

**DOI:** 10.3390/antibiotics5040038

**Published:** 2016-12-13

**Authors:** Roberto Chulluncuy, Carlos Espiche, Jose Alberto Nakamoto, Attilio Fabbretti, Pohl Milón

**Affiliations:** 1 Centro de Investigación e Innovación, Faculty of Health Sciences, Universidad Peruana de Ciencias Aplicadas—UPC, Lima L-33, Peru; robertochulluncuy1@gmail.com (R.C.); carlosespiche852@gmail.com (C.E.); jose.nakamoto@upch.pe (J.A.N.); 2 Facultad de Ciencias y Filosofía Alberto Cazorla Talleri, Universidad Peruana Cayetano Heredia—UPCH, Lima L-31, Peru; 3 Laboratory of Genetics, Department of Biosciences and Veterinary Medicine, University of Camerino, 62032 Camerino, Italy; attilio.fabbretti@unicam.it

**Keywords:** streptomycin, kanamycin, translation initiation, 30S subunit, IF3, tuberculosis, FRET

## Abstract

Aminoglycoside antibiotics are widely used to treat infectious diseases. Among them, streptomycin and kanamycin (and derivatives) are of importance to battle multidrug-resistant (MDR) *Mycobacterium tuberculosis*. Both drugs bind the small ribosomal subunit (30S) and inhibit protein synthesis. Genetic, structural, and biochemical studies indicate that local and long-range conformational rearrangements of the 30S subunit account for this inhibition. Here, we use intramolecular FRET between the C- and N-terminus domains of the flexible IF3 to monitor real-time perturbations of their binding sites on the 30S platform. Steady and pre-steady state binding experiments show that both aminoglycosides bring IF3 domains apart, promoting an elongated state of the factor. Binding of Initiation Factor IF1 triggers closure of IF3 bound to the 30S complex, while both aminoglycosides revert the IF1-dependent conformation. Our results uncover dynamic perturbations across the 30S subunit, from the A-site to the platform, and suggest that both aminoglycosides could interfere with prokaryotic translation initiation by modulating the interaction between IF3 domains with the 30S platform.

## 1. Introduction

Bacterial pathogens account for 38% of human infections [1] and, because of their potential to develop antibiotic resistance, represent a severe threat to human health. The problem is of particular importance in underdeveloped countries, where the incidence of multidrug-resistant (MDR) and extensively drug-resistant (XDR) mycobacteria and bacteria is rapidly increasing (World Health Organization, WHO). Tuberculosis (TB), caused by *Mycobacterium tuberculosis*, is a devastating disease with higher incidence in underdeveloped countries than in their developed counterparts ([2] and references therein). Antibiotics are the only option to treat TB efficiently. Streptomycin along with kanamycin and its derivative Amikacin are used as second-line drugs for the treatment of MDR tuberculosis [3,4].

**Figure 1:**
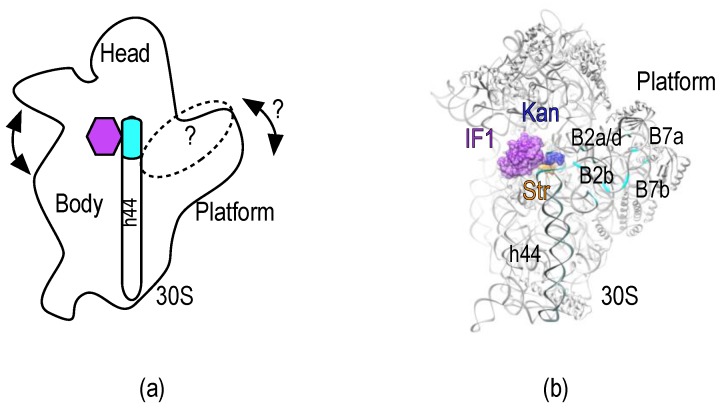
30S subunit, dynamic domains, and inter-subunit bridges. (**a**) Representation of the 30S subunit as seen from the 50S-interacting side. The main dynamic domains of the small subunit are indicated. Arrows represent the known and potential movements involved in mistranslation of the mRNA. The purple hexagon indicates the decoding center and binding site of IF1, streptomycin and kanamycin. The dotted oval indicates the overall binding surface of IF3 on the 30S platform; (**b**) Crystal structure of the IF1-30S subunit complex (Purple surface, PDB: 1HR0) [13]. Streptomycin (orange) and kanamycin (blue) were aligned from PDB: 4DR3 and PDB: 2ESI, respectively [14,15]. Cyan ribbons highlight residues involved in inter-subunit bridges of the 30S platform that overlap with IF3 binding sites.

Kanamycin and Streptomycin bind the decoding site (A-site) of the minor ribosomal subunit (30S) and inhibit protein synthesis mainly by causing misreading of the mRNA [5] or translocation inhibition (Kanamycin) [6,7]. The streptomycin-resistant strains contain hyper-accurate ribosomes [8]. During decoding the 30S subunit samples various conformations (Figure 1a) and the accuracy of the process can be affected by favoring a particular 30S state [9]; it can be surmised that streptomycin increases the misreading insofar as it promotes a ribosomal conformation that decreases the decoding accuracy.

Due to streptomycin’s effects, it was proposed that this drug could trigger an “error catastrophe” during protein elongation [10]. However, this model is in conflict with bot, the observation that ribosomes isolated from streptomycin-treated cells do not differ in speed and accuracy from those isolated from untreated *E. coli* cells [11], and that streptomycin causes 70S monomer accumulation and polysome depletion in vivo [12].

Streptomycin, kanamycin and initiation factor IF1 bind nearby within the decoding center of the 30S subunit and promote diverse local and long range conformational perturbations [13,14,15,16,17]. IF3 binds to the 30S platform, making contacts with h45, h23, and h24 of the 16S rRNA and ribosomal proteins uS7 and uS11 [18,19,20,21,22]. IF3 is a basic protein constituted by two globular domains of similar masses, N-terminal (NTD) and C-terminal (CTD), connected by a flexible linker [23,24]. The two domains are separated by a hydrophilic, lysine-rich flexible linker. Results of NMR spectroscopy, neutron scattering, mutagenesis, and accessibility to proteolysis indicate that IF3 NTD and CTD move independently [25,26,27]. Furthermore, real-time probing experiments [19] have demonstrated that IF3 CTD is the first to contact the 30S platform, immediately followed by the IF3 NTD. Interestingly, streptomycin binding was found to increase the dissociation rate of IF3 from non-canonical 30S initiation complexes (IC) [28].

The 30S platform greatly contributes to the association of the small subunit with the major ribosomal subunit (50S) through the formation of several inter-subunit bridges (Figure 1). The interaction of IF3 with the 30S subunit lays across the platform and regulates the progression of the 30S IC towards elongation of protein synthesis [19,20]. Here, we specifically labeled each domain of IF3 with fluorescent dyes (IF3_DL_) to develop an intramolecular Förster resonance energy transfer (FRET) system capable of sensing rapid conformational changes of the factor and/or its binding sites at the 30S platform. In combination with pre-steady state kinetics, the FRET signal of IF3_DL_ responds to the interaction of streptomycin, kanamycin, and IF1 with the A-site of the 30S subunit. Our data, in combination with recent structural studies, suggest a novel molecular mechanism for the aminoglycosides as capable of perturbing IF3 binding sites on the 30S platform.

## 2. Results

### 2.1. Experimental Outline

**Figure 2:**
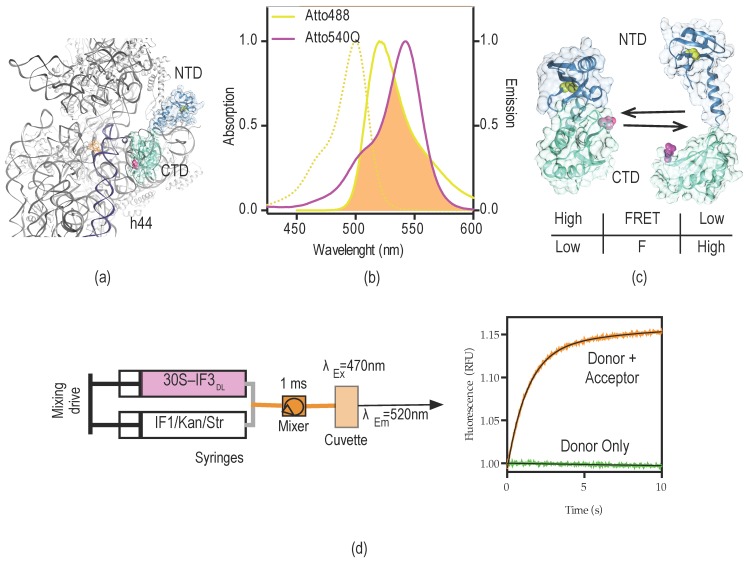
IF3_DL_ intramolecular FRET for dynamic measurements of the 30S platform. (**a**) Crystal structure of the 30S subunit, depicting a possible orientation of IF3 across the platform, CTD (turquoise), and NTD (steel blue); (**b**) absorption and emission spectra of Atto-488 fluorescent dye (yellow) and Atto-540Q quencher (purple). The overlap area between donor emission fluorescence (Atto-488) and acceptor absorption of quencher (Atto-540Q) is indicated in orange. R_0_ distance for the FRET couple is 64 Å according to the producer (Atto-tec, Siegen, Germany); (**c**) Potential arrangements of IF3 domains with respect to each other. Colors are as in (**a**); cysteine residues for donor (C65, yellow) and acceptor (E166C, purple) dyes are shown as spheres. The bottom table indicates the possible readouts of fluorescence and FRET for the corresponding states of IF3_DL_; (**d**) Scheme of stopped-flow experimental set-up and the typical signal read out upon mixing 30S–IF3_DL_ with a 30S binder (orange trace). In order to assign the signal as FRET, the same experiment is performed in the absence of the acceptor, in this case IF3_NAtto488_, IF3 labeled at the natural cysteine in the NTD (green trace).

IF3 binds across the 30S platform, interacts with several intersubunit bridges, and responds to conformational states of the small subunit (Figure 1) [17,19,20,28,29]. Therefore, IF3 could be used as sensor of structural changes occurring in the 30S platform. The structure of IF3 NTD consists of a globular α/β fold, constituted by a four-stranded b-sheet onto which an α-helix is packed [30]. IF3 CTD is composed by a two-layered α/β sandwich fold with a βαβαββ topology with two parallel α-helices packed against a four-stranded β-sheet [23] (Figure 2a,c). Naturally, IF3 contains a sterically buried single cysteine at position 65 of the NTD which reacts slowly with maleimide moieties [31,32]. We introduced a second cysteine at a solvent-exposed position of the CTD (E166C) to kinetically enhance the fluorescent modification of the CTD over the NTD. Aiming to obtain a very sensitive intramolecular FRET system, Atto-488 and Atto-540Q were chosen as the fluorescence donor and non-emitting acceptor (quencher), respectively.

The R_0_ between the dyes is Å 64, providing a wide range of distances to be monitored by changes of FRET efficiencies (Figure 2b). Under native buffer conditions IF3_E166C_ reacted efficiently with Atto-540Q maleimide (Figure S1c). In order to enhance the poor reactivity of C65 at the NTD, IF3_E166C_–Atto540Q was subsequently modified with Atto-488 maleimide under denaturing conditions. Finally, the resulting doubly labeled protein (IF3_DL_) contained a non-emitting acceptor (quencher) at the CTD and a fluorescent dye at the NTD (Figure S1c) (see Materials and Methods for details). Therefore, the vicinity of the dyes (domains) would result in donor fluorescence quenching, while the opposite would increase the observed fluorescence. A high FRET state corresponds to low fluorescence read-outs, indicating the vicinity of IF3 domains with respect to each other (Figure 2c).

### 2.2. Probing the Sensing Limits of IF3_DL_

IF3 and IF1 cooperatively increase their affinity for the 30S subunit [21,33]. Along the pathway of translation initiation, both factors rapidly join the 30S subunit concomitantly with IF2. The whole process of 30S pre-Initiation Complex (pre-IC) formation takes around 100 ms and precedes fMet-tRNA^fMet^ (initiator tRNA) recruitment [33,34]. This multi-component process can follow multiple pathways, as shown by single molecule measurements [35]. The cooperation between IF1 and IF3 is suggested to maintain the fidelity of translation initiation; however, its molecular dynamics remain elusive [28,36].

**Figure 3:**
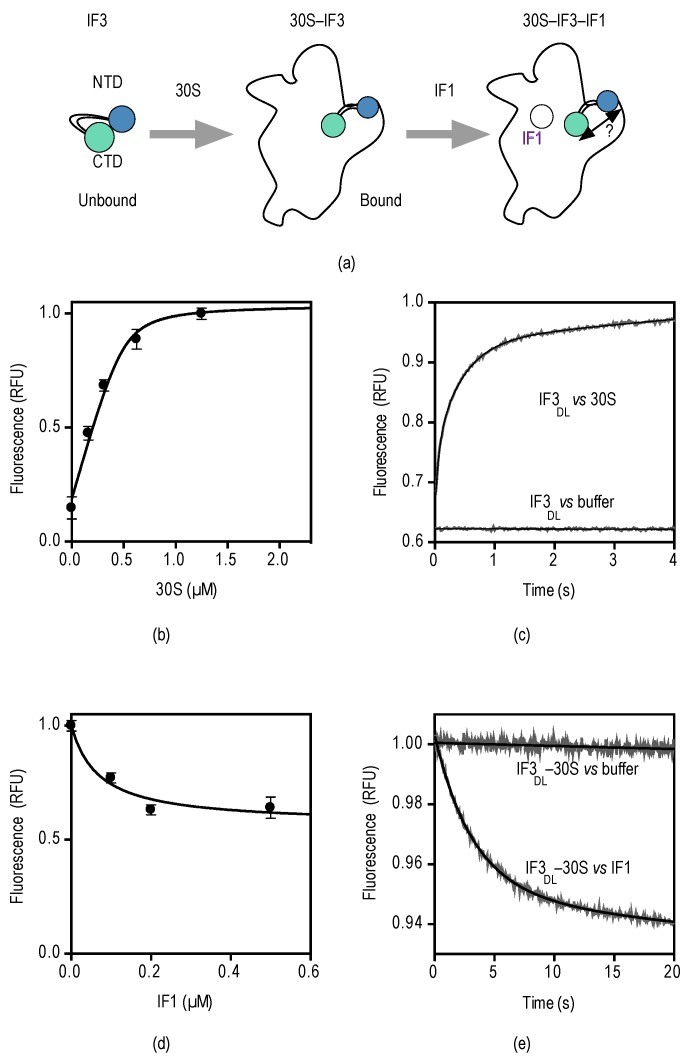
Steady and pre-steady binding of IF3_DL_ and IF1 to the 30S subunit. (**a**) Experimental scheme depicting the binding reactions of IF3 and IF1; (**b**) IF3_DL_ titration with increasing concentrations of 30S subunits. IF3_DL_ (0.5 µM) was incubated with the indicated concentrations of 30S subunits for 10 min. 5 replicates of 2 µL were measured in a NanoDrop 3000 fluorimeter (Thermo Fisher Scientific, Waltham, MA, USA). Error bars indicate standard deviations (SD). Continuous line shows fitting with a quadratic equation for binding (see Materials and Methods); (**c**) Time courses of IF3_DL_ binding to 30S subunits and a buffer control to assign the specific amplitude change. 30S subunits (0.1 µM) were mixed with equimolar IF3_DL_ in a stopped-flow apparatus. Ten to 12 individual traces were recorded and averaged. Smooth lines indicate fits by non-linear regression with two exponential terms; (**d**) 30S–IF3_DL_ (0.5 µM) titration with IF1 at the indicated concentrations. Five replicates of 2 µL were measured as above. Error bars indicate standard deviations (SD); (**e**) Time courses of 30S–IF3_DL_ binding to IF1 and a buffer control to assign the specific amplitude change. See Figure S3 for no acceptor controls. 30S–IF3_DL_ complexes (0.1 µM) were mixed with a 10-fold molar excess of IF1 (1 µM) in a stopped-flow apparatus. Twelve individual traces were recorded and averaged. Smooth lines indicate fits by non-linear regression with a single exponential term.

Here, we measure the binding kinetics of IF3_DL_ to the 30S subunit and the influence of IF1 in the resulting 30S–IF3_DL_ complex (Figure 2d). NMR measurements of full-length IF3 indicated that its domains can freely move in solution, adopting almost random orientations [25]. However, molecular modeling and site-directed mutagenesis proposed that IF3 can transiently establish inter-domain contacts [27]. In any case, the transition from unbound to bound to the 30S subunit would result in IF3 adopting an elongated conformation on the 30S platform [19,20,37] (Figure 3a).

Indeed, fluorescence equilibrium measurements of IF3_DL_ titrations at increasing concentrations of 30S subunits (0.2–2.5 µM) resulted in proportional increased emission fluorescence (Figure 3b), indicating that IF3 transits towards an extended open state in the 30S platform. Fitting of the measurements with a quadratic function for binding kinetics yielded a dissociation constant *K_D_* for the 30S–IF3 complex in the low nanomolar range, consistent with previous pre-steady state measurements [33]. Binding of IFs to the 30S subunit and their subsequent conformational rearrangements are rapid, taking place in few seconds. Therefore, to measure the pre-steady state binding of IF3_DL_ to the 30S subunit we rapidly mixed 30S subunits with IF3_DL_ in a stopped-flow apparatus (KintekCorp, Snow Shoe, PA, USA) and measured fluorescence emission after passing a 515 nm long-pass optical filter.

Upon mixing of IF3_DL_ with 30S subunits, the fluorescence increased with time with a biphasic behavior (Figure 3c). In contrast, a control where the factor was mixed with buffer or lacked the acceptor at the CTD did not show a change of fluorescence (Figure S3). Non-linear regression fitting of the recorded measurements with an equation containing two exponential terms yielded two apparent rate constants, *k*_app1_ and *k*_app2_, and two associated fluorescence amplitudes, F_1_ and F_2_ (Equation (2)). Analysis is consistent with an initial bimolecular encounter between IF3_DL_ and the 30S subunit, *k*_app1_ = 16 ± 1 s^−1^, followed by a conformational rearrangement of the factor, *k*_app2_ = 2 ± 0.05 s^−1^. These measurements are in agreement with previous rapid kinetic studies, although different fluorescent reporters, 30S subunits, and IFs purification methods were used [33,38].

Then we investigated whether IF3_DL_ pre-bound to the 30S subunit could monitor IF1 interactions with the ribosome. Equilibrium titrations of IF3_DL_–30S with increasing concentrations of IF1 resulted in a decrease of emitted fluorescence, indicating that IF3 domains reach closer distances, consistent with previous single-molecule studies [37] (Figure 3d). Fitting of the measurements for one-site binding yielded a *K_D_* of about 100 nM, consistent with measurements by pre-steady state methods [33].

Equilibrium measurements of the interaction of IF1 with the 30S–IF3_DL_ complex indicated a decrease in distance between the domains of IF3. We then explored whether the kinetics of the interaction would reflect a bimolecular encounter of IF1 with the complex or a later conformational rearrangement. Preformed 30S–IF3_DL_ complexes were rapidly mixed with a 10-fold molar excess of IF1 in a stopped-flow apparatus and fluorescence was measured with time as described above. Upon mixing, the fluorescence of IF3_DL_ decreased exponentially and was best described by a single exponential term equation (Equation (4)) (Figure 3e). Fitting of the measurements yielded an apparent rate constant *k*_app_ = 0.26 ± 0.01 s^−1^. Previous studies reported an association constant for the bimolecular encounter of IF1 with the 30S–IF3 complex of 20 µM^−1^·s^−1^ [33], thus we expected an apparent rate for IF1 of ≈20 s^−1^ (IF1 = 1 µM). Then, IF3_DL_ reports an IF1-dependent FRET change that is ≥75-fold slower than the initial binding, suggesting that IF3_DL_ is monitoring a successive step, i.e., a conformational rearrangement of IF3 on the platform resulting in the accommodation of one of IF3 domains.

IF3_DL_ allows monitoring equilibrium and real-time kinetics of factor binding, dissociation, as well as conformational changes induced by IF1. The intramolecular FRET sensibility and versatility of IF3_DL_ provide solid bases to test 30S subunit binders, with a special emphasis for those targeting the A-site. Among them, streptomycin and kanamycin stood out because of their medical importance as second-line drugs to treat MDR TB.

### 2.3. Counter Effects between IF1 and Streptomycin/Kanamycin

**Figure 4:**
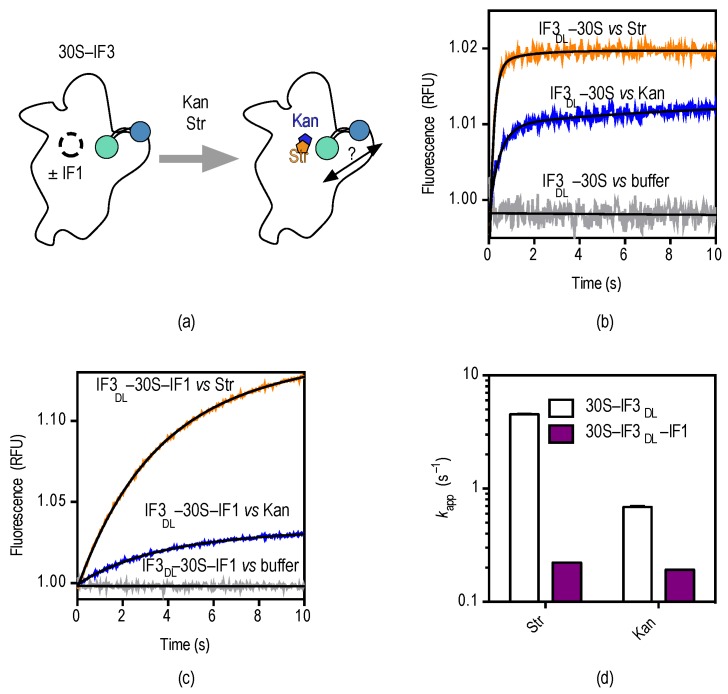
Pre-steady state kinetics of streptomycin and kanamycin binding to 30S–IF3_DL_ complexes. (**a**) Scheme depicting the experimental approach to monitoring conformational effects as a function of streptomycin (orange) and kanamycin (blue); (**b**) time courses of 30S–IF3_DL_ (0.1 µM) interacting with each aminoglycoside; colors are as in (**a**). The trace for buffer control indicates no dissociation of IF3_DL_ during rapid mixing in the stopped-flow apparatus. See Figure S3 for controls in the absence of fluorescence acceptor; (**c**) Time courses of streptomycin and kanamycin binding to 30S–IF3_DL_–IF1 (0.1 µM). Smooth lines indicate fits by non-linear regression (Equation (4)); (**d**) Influence of IF1 over the kinetics of IF3_DL_ conformational changes caused by streptomycin and kanamycin.

The conformational cooperation between IF1 and IF3 observed above has been suggested to maintain the fidelity of translation initiation [28,36]. On the other hand, streptomycin was shown to disrupt the cooperation between the factors, and possibly overall fidelity, by increasing the velocity of formation of 70S IC programmed with non-canonical mRNAs [28]. Here, we use 30S–IF3_DL_ complexes to monitor real-time conformational perturbations of IF3 on the platform upon the binding of streptomycin and kanamycin to the A-site (Figure 4a).

Rapid mixing of either aminoglycoside with 30S–IF3_DL_ complexes in a stopped-flow apparatus results in an exponential increase of fluorescence over time, indicating that IF3 domains get further apart (Figure 4b). The measurements were best described by a single exponential term yielding an apparent rate (*k*_app_) and an associated fluorescent amplitude (F) (Equation (4)). Analysis by non-linear regression fitting returned apparent rates for streptomycin and kanamycin *k*_app_^Str^ = 4.6 ± 0.1 s^−1^ and *k*_app_^Kan^ = 1.4 ± 0.1 s^−1^ (Figure 4d).

Streptomycin and kanamycin have opposite effects on the 30S platform if compared to IF1 as observed by IF3_DL_. While IF1 closes up IF3 domains, the aminoglycosides bring them apart. Each A-site binder is also characterized by different extents of FRET change with IF1 promoting an opposite and greater (≈3-fold) perturbation if compared to the aminoglycosides. Consequently, we probed whether the interaction of streptomycin and kanamycin with the 30S subunit could revert IF1-dependent closing up of IF3_DL_ (Figure 4c).

**Figure 5:**
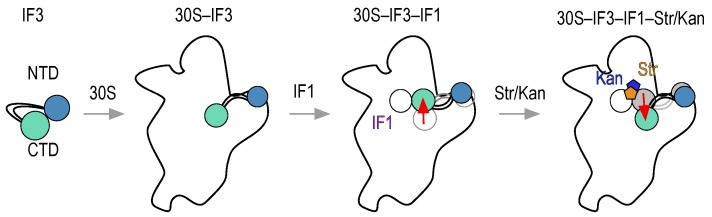
Scheme of IF3 movements during early stages of translation initiation and response to antibiotics streptomycin and kanamycin. IF3 binding to the 30S subunit results in opening of the factor and adopting an overall elongated state. The interaction of IF1 (purple) results in repositioning of IF3, probably shifting the CTD (magenta) towards the P-site and possibly interacting with IF1. Streptomycin (orange) and kanamycin (blue) would perturb the equilibrium between binding sites of IF3, promoting a displacement of the factor. Red arrows indicate possible movements of IF3. Gray shadows indicate IF3 states prior to the interaction of the binder with the 30S subunit.

Rapid mixing of 30S–IF3_DL_–IF1 complexes with either streptomycin or kanamycin in a stopped-flow apparatus resulted in an exponential increase of fluorescence (Figure 4c). Comparisons of the drugs binding to 30S–IF3_DL_ complexes (without IF1) showed an increased amplitude of fluorescence change and slower apparent rates (Figure 4d). Nonlinear fitting of the time dependencies with a single exponential function (Equation (4)) indicated a 20-fold and 7-fold decrease of the *k*_app_ for streptomycin and kanamycin in the presence of IF1, respectively (Figure 4d). On the contrary, the amplitudes of FRET changes were increased in the presence of IF1. Thus, kanamycin and streptomycin seem to compete with IF1, imposing an IF3 layout on the platform similar to that in 30S complexes lacking IF1 (Figure 5). In addition, reversion of the IF1-dependent conformation shows similar apparent rates for both aminoglycosides (*k*_app_^Str^ = 0.2 ± 0.01 s^−1^ and *k*_app_^Kan^ = 0.22 ± 0.01 s^−1^), suggesting they are rate-limited by a similar reaction.

The intrinsic flexibility and dynamics of IF3 seem to sample different conformational states of the 30S subunit, at the 30S platform where the factor binds. With opposing directions, IF1 together with streptomycin and kanamycin alter the relative disposition of IF3 domains, revealing molecular mechanisms of antibiotic action at an unexpected site but with potential functional implications. Perturbing IF3 binding sites by streptomycin, even in the presence of IF1, provides a rationale to previous reports where the absence of IF1 or the addition of streptomycin increased the rates of non-canonical translation initiation [28].

## 3. Discussion

Both, streptomycin and kanamycin, disturb the positioning of IF3 at the 30S platform, possibly affecting translation initiation (Figure 4) in addition to later steps of translation. It is generally accepted that streptomycin and kanamycin inhibit cell growth by increasing mRNA misreading during elongation of protein synthesis (reviewed in [39]). This notion is derived from polyU directed poly-Phe synthesis experiments where the drugs induced mis-incorporation of other amino acids into the peptide chain [40]. In support, streptomycin caused phenotypic suppression of nonsense mutations in vivo [41]. More recent biochemical, structural, and single-molecule studies strengthen the notion of streptomycin and kanamycin (and other aminoglycosides) affecting decoding, elongation, and translocation [39,42,43]. On the initiation side, streptomycin increased the velocity of 70S IC formation if programmed with non-canonical mRNAs, suggesting that the aminoglycoside could cause loss of translation initiation fidelity [28]. The effect was associated with an increase of IF3 dissociation rate through a conformational switch at the 30S subunit. Streptomycin would weaken the binding sites of IF3 at the platform, therefore increasing premature 50S joining. Thus, streptomycin would result in *in vivo* formation of unproductive 70S complexes. This postulation is supported by experiments from the late 1960s which indeed showed streptomycin to cause an accumulation of 70S monomers concomitantly to a reduction of polysomes, consistent with streptomycin preferentially inhibiting early steps of translation [12,44].

We observe that IF1 binding decreases the donor fluorescence of IF3_DL_ (increased FRET), interpreted as a closing up of IF3 domains (Figure 3). IF1 was shown to bind away (>50 Å) from either domain of IF3, suggesting that the closing up of IF3 is rather indirect, through an allosteric effect of IF1 across the 30S subunit [21]. However, recent structural studies show that each domain of IF3 can occupy at least two positions on the 30S subunit as a function of ligands bound to the initiation complex [18]. The CTD seem to contact IF1 in complexes lacking initiator tRNA. In full 30S ICs the CTD positions under the tRNA, moving away from its initial position. Also the NTD of IF3 was shown to interact nearby uS11, at the tip of the platform, or to initiator tRNA in full complexes. In a lesser extent, single molecule approaches observed similar dynamics of IF3. Specifically, the effect of IF1 on IF3 layout was shown to transit away from an extended conformation towards a more closed state [37].

Functionally, rapid kinetic and biochemical assays showed a close relationship between IF3 and the mRNA in an IF1-dependent manner. IF3 can promote mRNA shift and can indirectly discriminate unfit mRNAs, i.e., non-canonical codons [28,45,46,47]. The crosstalk between IF3 and IF1 is also supported by several isolated mutations, which increased translation initiation from non-canonical codons, clustered in the 790 loop (interacting with IF3) and h44 (at residues known to be distorted by IF1).A cooperation between IF1 and IF3 enhances the fidelity of translation initiation [36].

Consistently, IF1 increases IF3 affinity for the 30S subunit in a cooperative manner [33]. Omission of IF1 resulted in an increased premature 70S IC formation, a similar effect obtained in the presence of streptomycin [28]. Thus, streptomycin and IF1 would favor opposite states of IF3 on the 30S subunit. The closer distances between IF3 domains observed in this study would represent a 30S subunit with the most 50S anti-association property. On the other hand, a more open state of the factor would facilitate the arrival of the major subunit. Streptomycin and kanamycin promote the opening of the factor (this study, Figure 4) and streptomycin increases the speed of IF3 dissociation and subunit association [28].

Streptomycin would perturb initiation of protein synthesis by reverting a high-fidelity IF3 layout on the 30S subunit that is induced by IF1. Consequently, the overall initiation fidelity threshold is lowered by the aminoglycoside, allowing premature joining of the major subunit. Our results expand the range of reactions that aminoglycosides may affect and provide insights into the dynamic molecular network that they exploit. Streptomycin stabilizes the pairing of A1413-G1487 as it hinders G1487 from kethoxal modification [48] (Figure S4). Additionally, streptomycin is proposed to cause conformational changes in the h45 tetraloop. This loop was shown to adopt two different states, called “engaged and disengaged”, with respect to h44 (nucleotide C1496), with streptomycin favoring the disengaged state [14]. In this state the h45 tetraloop moves away from the h44, with G1517 swinging counter-clockwise about 5 Å away (Table S1 and Figure S4). Streptomycin interacts with G1491, not affecting A1492 and A1493. On the contrary, IF1 flips out both residues (1492–1493), promotes the engaged state between h44 and h45 (C1496-G1517), and unpairs A1413 and U1414 from G1487 and G1486, respectively [13] (Figure S4). Altogether, streptomycin promotes opposite to IF1 structural changes across three directions, towards the tip of h44, downstream h44, and towards the platform, through the h44/h45 interaction (Table S2 and Figure S4).

Thus, IF1 and streptomycin seem to exploit the same structural network, yet in opposite directions (Figure 5). Our results indicate that IF1 and streptomycin/kanamycin also display opposite effects for the inter-domain distance of IF3, with the antibiotics increasing the distance while IF1 decreases it. These counter effects may find a rationale in the structural network described above, where different residues of the 30S subunit may be exposed for preferential binding of IF3. As observed by cryoEM, IF3 domains can bind to different sites on the 30S platform [18]. Our results may indicate that IF1, streptomycin, and kanamycin perturb the equilibrium of the CTD between its two binding sites. A direct interaction of IF1 with the CTD of IF3 may contribute to enhance the close state of IF3 observed here.

Structural and kinetic analysis show that the engaged/disengaged state of the 30S subunit is also affected by the novel antibiotic GE81112, resulting in a blockade of the 30S IC progression by preventing initiation codon decoding [49]. Whether GE81112 perturbs the IF3 layout of the 30S subunit remains elusive; however, our model would suggest that the drug promotes a close distance conformation between domains. Conformational changes that are sensed by IF3 in the platform area may also affect the association of the 30S with the 50S through differential exposure of intersubunit bridges (Figure 1b). Besides B2b, which is collocating with the IF3 binding site, B7a–b may be regulated as in different rotate states of the ribosome. Altogether, the dynamic platform could provide a rationale for the tight regulation of the anti-association function of IF3 as modulating the accessibility of each domain for their binding sites.

Finally, the biophysical system depicted in this work can be used as a novel platform to identify and characterize compounds targeting initiation of translation [50]. Indeed, screening systems to identify compounds that preferentially inhibit the initiation phase have proved successful [51,52,53]. In addition, our IF3_DL_-30S reporter assay can provide novel aspects of the inhibiting mechanism of known 30S-binding drugs. Similar approaches have allowed detailed descriptions for other inhibitors of the ribosome [54,55].

## 4. Materials and Methods

### 4.1. Escherichia coli Strains, Expression Vectors, Cell Growth, and Protein Expression Induction

Competent *E. coli* BL21_DE3_ cells were CaCl_2_ transformed (Mix & Go, Zymo Research, Irvine, CA, USA) with either expression vector pET24c *Inf*A, pET24c *Inf*C *wt*, or pET24c *Inf*C E166C, coding for IF1, IF3 *wt*, or IF3_E166C_, respectively. pET24c vectors containing *wt* and mutant genes were commercially acquired (GenScript, Piscataway, NJ, USA). Typically, 2 L of Luria–Bertoni (LB) medium were used to grow BL21_DE3_ pET24c *Inf*A or pET24c *Inf*C to an OD_600nm_ of 0.5. Protein expression was induced by adding 1 mM Isopropyl β-d-1-thiogalactopyranoside (IPTG, Thermo Fisher Scientific). Cells were allowed to express IF1 or IF3 for 3 h prior to harvesting by centrifugation at 5000× *g* at 4 °C. Cells were lysed in Lysis Buffer (50 mM Hepes pH: 7, 100 mM NH_4_Cl, 10 mM MgCl_2_, 10% Glycerol, 6 mM 2-mercaptoetanol) supplemented with 0.1 mg/mL of Lysozyme (Merck, Darmstadt, Germany). After five cycles of freezing and thawing, 1 U/mL DNAse I was added to reduce the viscosity in 20 min of incubation at 4 °C. Membranes and supernatant were separated by centrifugation at 15,000× *g* for 30 min.

### 4.2. IF1, IF3, and 30S Subunits Purification

Both initiation factors were purified by Cation exchange chromatography on HiTrap SP HP (Amersham, Uppsala, Sweden). Supernatants were manually loaded to the column (1 mL column volume) and subsequently subjected to a linear NH_4_Cl gradient (0.05–1 M) in a Jasco HPLC system (Jasco, Tokyo, Japan). The gradient was prepared in Buffer_A_ (50 mM Hepes pH 7.1, 10% Glycerol, 6 mM 2-Mercaptoethanol). IF3 and IF1 were eluted at 700 mM and 400 mM of NH_4_Cl, respectively, in (Figures S1a and S2a). The best separation conditions were 1 mL/min flow rate and 20 Column Volumes (CV) long gradient, collecting fractions of 1 mL each. Protein elution was followed by absorbance at 290 nm and SDS-Polyacrylamide Gel Electrophoresis (SDS-PAGE, 15%) (Figures S1 and S2). While IF3 was eluted with an elevated degree of purity, IF1 fractions contained high molecular weight contaminates (Figure S2b). Full elimination of the contaminants was obtained by subjecting the combined IF1 fractions to Amicon^®^ Ultra 30K Da centrifugal filters (Merck) followed by concentration on a HiTrap SP HP (Amersham), single step eluted with 1 M NH_4_CL Buffer_A_ (Figure S2c).

30S subunits purification methods are described in detail in [38].

### 4.3. Double Labeling of IF3 with Atto-Tec Dyes

IF3_E166C_ was subjected to extensive dialysis in labeling buffer (50 mM Hepes pH: 7.1, 100 mM NH_4_Cl, 10% glycerol, 0.5 mM TCEP) in a D-Tube™ Dialyzer Maxi (Merck) to remove traces of 2-mercaptoethanol as the reducing agent strongly inhibits the coupling of maleimide-linked dyes to cysteines. First, the C-terminal was labeled at the recombinant cysteine (166) as it is exposed and efficiently reacts with maleimide derivatives [38]. A 10-fold excess of Atto-540Q maleimide (Atto-Tec) over IF3_E166C_ was incubated in labeling buffer for 20 min. The reaction was stopped by the addition of 6 mM 2-mercaptoethanol. The modified IF3_CTD_^540Q^ was purified from unreacted dyes on a HiTrap SP HP column. After 10 CV washes with Buffer_A_ containing 100 mM NH_4_Cl, a single step elution was applied using 3 mL of 1 M NH_4_Cl in Buffer_A_. Typically, full protein recovery is achieved in 0.5 mL and elution of the labeled protein is readily visible. IF3 _CTD_^540Q^ was subsequently dialyzed as mentioned above in a labeling buffer containing 2 M UREA.

Denaturation of IF3 results in the exposure of the otherwise buried cysteine at position 65 of the NTD. The denatured protein was incubated with a 10-fold molar excess of Atto-488 maleimide for 1 h at RT, mild shacking was applied. IF3_CTD_^540Q^_–NTD_^488^ (IF3_DL_) was purified from the unreacted dye as described above using HiTrap SP HP column (Merck). Eluted proteins were dialyzed against storage buffer (Hepes pH: 7.1, 100 mM NH_4_Cl, 10% Glycerol, 6 mM 2-mercaptoethanol) and small aliquots were stored at −80 °C. Purity and efficiency of labeling was assayed by 15% SDS-PAGE, where fluorescence was observed under a UV trans-illuminator and total protein by blue Coomassie staining (Figure S1c).

### 4.4. Equilibrium Binding Measurements

All reactions were performed in HAKM_10_ buffer (50 mM HEPES 70mM, NH_4_Cl, 30 mM KCl, 10 mM MgCl_2_, 6 mM 2-Mercaptoethanol). 30S titrations of IF3_DL_ (0.5 µM) were incubated with varying concentrations of 30S subunits (0.16, 0.3125, 0.625, 1.25, 2.5 µM). Reactions were incubated for 10 min at 37 °C. Fluorescence was measured in a NanoDrop 3000 fluorimeter (Thermo) using blue LED excitation and emission at maximum for Atto-488 (518 nm) at room temperature. Typically, five independent measurements were performed for each reaction to calculate mean and standard deviation values. Binding of IF1 to 30S–IF3DL complexes was performed as above after pre-incubating IF3_DL_ with 30S subunits for 10 min at 37 °C. IF1 influence was measured at varying concentration of the factor (0.1, 0.2, 0.5, 1, 2 µM). 30S subunits were MgCl_2_ (20 mM) activated for 30 min at 42 °C prior to being used.

### 4.5. Stopped-Flow Measurements and Analysis

Fluorescence stopped-flow measurements were performed using a SF-300X stopped-flow apparatus (KintekCorp) by rapidly mixing equal volumes (30 µL each) of reacting solutions (Figure 2a). Excitation wavelength for Atto-488 was 470 nm and emission was measured after a long-pass optical filter with a 515 nm cut-off. One thousand points were acquired in 20–30 s of each measurement. Ten to 15 replicates were recorded for each reaction and subsequently averaged. All stopped flow reactions were performed in TAKM_10_ buffer (50 mM Tris (pH: 7.5), 70 mM NH_4_Cl, 30 mM KCl, 10 mM MgCl_2_, 6 mM 2-Mercaptoethanol) at 25 °C; 30S and IFs concentrations are given in the figure legends.

### 4.6. Data Analysis

Non-linear regressions by Prism 6.0 (Graphpad Software, La Jolla, CA, USA) were performed using the following equations:

(1) $$\left[C\right]=\frac{\left(\left[A\right]+\left[B\right]+K_{D}\right)-\;\sqrt{{\left(\left[A\right]+\left[B\right]+K_{D}\right)}^{2}-4\left[A\right]\left[B\right]}}{2},$$

with A = 30S; B = IF3_DL_; C = 30S–IF3_DL_ and *K_D_* is the dissociation constant.

(2) $$F=F_{0}+F_{1}e^{kapp1\;\times \;t}+F_{2}e^{kapp2\;\times \;t}$$

(3) $$\left[C\right]=\frac{\left[A\right]\left[B\right]}{K_{D}+\left[B\right]};$$

A = 30S–IF3_DL_; B = IF1; C = 30S–IF3_DL_–IF1.

(4) $$F=F_{0}+F_{1}e^{kapp1\;\times \;t}$$

### 4.7. Structural Models

Molecular models were derived from the structures of 30S–bound IF1 of *Thermus thermophiles* (PDB 1HR0; [13]), streptomycin bound to the 30S (PDB 4DR3; [14]), kanamycin bound to the site-A section of h44 (PDB 2ESI; [15]) and the apo-30S subunit (PDB 4DR1; [14]) (Table S3). The structural models showing the binding site of IF1, streptomycin and kanamycin where generated by aligning the structures through the backbone atoms of the 16S rRNA (Full sequence for 30S/IF1 and 30S/streptomycin and partial sequence for the A-site with kanamycin) using Chimera and Swiss PDB viewer [56,57]. Molecular graphics and analyses were performed with the UCSF Chimera package. Chimera is developed by the Resource for Biocomputing, Visualization, and Informatics at the University of California, San Francisco (supported by NIGMS P41-GM103311). Through this work the new nomenclature for ribosomal proteins has been used [58].

## Supplementary Materials

The following are available online at http://www.mdpi.com/2079-6382/5/4/38/s1, Table S1: Distances from h44 to h45 of residues in the engaged state, disengaged state, and IF1-bound structures, Table S2: Summary of structural counter effects between streptomycin and IF1 on the 30S subunit, Table S3: Structures used for modeling. Figure S1: Purification and fluorescence labeling of IF3_E166C_, Figure S2: Purification of IF1, Figure S3: FRET controls for IF3_DL_ sensing of A-site binders, Figure S4: Possible structural changes induced by streptomycin and IF1 on the 30S subunit.

## Abbreviations

The following abbreviations are used in this manuscript:

MDR	Multidrug-resistant
XDR	Extensively drug-resistant
TB	Tuberculosis
IF3	Initiation factor 3
IF1	Initiation factor 1
CDC	Centers for Disease Control and Prevention
30S	Minor ribosomal subunit
IC	Initiation complexes
SD	Shine–Dalgarno sequence
NTD	N-terminal domain
CTD	C-terminal domain
FRET	Fluorescence Resonance Energy Transfer
IF3_DL_	Double-labeled IF3
Pre-IC	Pre-initiation complex
Initiatior tRNA	fMet-tRNA^fMet^
K_D_	Dissociation constant
k_app_	Apparent rate constant
SEM	Standard error of the mean
F	fluorescent amplitude
LB	Luria–Bertoni medium
IPTG	Isopropyl β-d-1-thiogalactopyranoside
SDS-PAGE	Sodium-dodecyl-sulfate-Polyacrylamide Gel Electrophoresis
HEPES	4-(2-hydroxyethyl)-1-piperazineethanesulfonic acid
TCEP	tris(2-carboxyethyl)phosphine

## References

[1] Taylor L.H., Latham S.M., Woolhouse M.E.J. (2001). Risk factors for human disease emergence. Philos. Trans. R. Soc. B Biol. Sci..

[2] Zumla A., Raviglione M., Hafner R., von Reyn C.F. (2013). Tuberculosis. N. Engl. J. Med..

[3] Blumberg H.M., Burman W.J., Chaisson R.E., Daley C.L., Etkind S.C., Friedman L.N., Fujiwara P., Grzemska M., Hopewell P.C., Iseman M.D. (2003). Centers for Disease Control and Prevention/Infectious Diseases Society of America: Treatment of tuberculosis. Am. J. Respir. Crit. Care Med..

[4] Horsburgh C.R., Feldman S., Ridzon R. (2000). Infectious Diseases Society of America Practice guidelines for the treatment of tuberculosis. Clin. Infect. Dis..

[5] Mingeot-Leclercq M.P., Glupczynski Y., Tulkens P.M. (1999). Aminoglycosides: Activity and resistance. Antimicrob. Agents Chemother..

[6] Pestka S. (1974). [28] The use of inhibitors in studies of protein synthesis. Methods Enzymol..

[7] Misumi M., Tanaka N. (1980). Mechanism of inhibition of translocation by kanamycin and viomycin: A comparative study with fusidic acid. Biochem. Biophys. Res. Commun..

[8] Gorini L., Jacoby G.A., Breckenridge L. (1966). Ribosomal ambiguity. Cold Spring Harb. Symp. Quant. Biol..

[9] Lodmell J.S., Dahlberg A.E. (1997). A conformational switch in *Escherichia coli* 16S ribosomal RNA during decoding of messenger RNA. Science.

[10] Blomberg C., Johansson J., Liljenström H. (1985). Error propagation in *E. coli* protein synthesis. J. Theor. Biol..

[11] Fast R., Eberhard T.H., Ruusala T., Kurland C.G. (1987). Does streptomycin cause an error catastrophe?. Biochimie.

[12] Luzzatto L., Apirion D., Schlessinger D. (1969). Streptomycin action: Greater inhibition of *Escherichia coli* ribosome function with exogenous than with endogenous messenger ribonucleic acid. J. Bacteriol..

[13] Carter A.P., Clemons W.M., Brodersen D.E., Morgan-Warren R.J., Hartsch T., Wimberly B.T., Ramakrishnan V. (2001). Crystal structure of an initiation factor bound to the 30S ribosomal subunit. Science.

[14] Demirci H., Murphy F., Murphy E., Gregory S.T., Dahlberg A.E., Jogl G. (2013). A structural basis for streptomycin-induced misreading of the genetic code. Nat. Commun..

[15] François B., Russell R.J.M., Murray J.B., Aboul-ela F., Masquida B., Vicens Q., Westhof E. (2005). Crystal structures of complexes between aminoglycosides and decoding A site oligonucleotides: Role of the number of rings and positive charges in the specific binding leading to miscoding. Nucleic Acids Res..

[16] Carter A.P., Clemons W.M., Brodersen D.E., Morgan-Warren R.J., Wimberly B.T., Ramakrishnan V. (2000). Functional insights from the structure of the 30S ribosomal subunit and its interactions with antibiotics. Nature.

[17] Moazed D., Samaha R.R., Gualerzi C.O., Noller H.F. (1995). Specific protection of 16S rRNA by translational initiation factors. J. Mol. Biol..

[18] Hussain T., Llácer J.L., Wimberly B.T., Kieft J.S., Ramakrishnan V. (2016). Large-scale movements of IF3 and tRNA during bacterial translation initiation. Cell.

[19] Fabbretti A., Pon C.L., Hennelly S.P., Hill W.E., Lodmell J.S., Gualerzi C.O. (2007). The real-time path of translation factor IF3 onto and off the ribosome. Mol. Cell.

[20] Dallas A., Noller H.F. (2001). Interaction of translation initiation factor 3 with the 30S ribosomal subunit. Mol. Cell.

[21] Julián P., Milon P., Agirrezabala X., Lasso G., Gil D., Rodnina M.V., Valle M. (2011). The Cryo-EM structure of a complete 30S translation initiation complex from *Escherichia coli*. PLoS Biol..

[22] Pon C.L., Pawlik R.T., Gualerzi C. (1982). The topographical localization of IF3 on *Escherichia coli* 30S ribosomal subunits as a clue to its way of functioning. FEBS Lett..

[23] Biou V., Shu F., Ramakrishnan V. (1995). X-ray crystallography shows that translational initiation factor IF3 consists of two compact alpha/beta domains linked by an alpha-helix. EMBO J..

[24] Garcia C., Fortier P.L., Blanquet S., Lallemand J.Y., Dardel F. (1995). Solution structure of the ribosome-binding domain of *E. coli* translation initiation factor IF3. Homology with the U1A protein of the eukaryotic spliceosome. J. Mol. Biol..

[25] Moreau M., de Cock E., Fortier P.L., Garcia C., Albaret C., Blanquet S., Lallemand J.Y., Dardel F. (1997). Heteronuclear NMR studies of *E. coli* translation initiation factor IF3. Evidence that the inter-domain region is disordered in solution. J. Mol. Biol..

[26] Gualerzi C.O., Pon C.L. (2015). Initiation of mRNA translation in bacteria: Structural and dynamic aspects. Cell. Mol. Life Sci..

[27] De Cock E., Springer M., Dardel F. (1999). The interdomain linker of *Escherichia coli* initiation factor IF3: A possible trigger of translation initiation specificity. Mol. Microbiol..

[28] Milon P., Konevega A.L., Gualerzi C.O., Rodnina M.V. (2008). Kinetic checkpoint at a late step in translation initiation. Mol. Cell.

[29] Hennelly S.P., Antoun A., Ehrenberg M., Gualerzi C.O., Knight W., Lodmell J.S., Hill W.E. (2005). A time-resolved investigation of ribosomal subunit association. J. Mol. Biol..

[30] Garcia C., Fortier P.L., Blanquet S., Lallemand J.Y., Dardel F. (1995). 1H and 15N resonance assignments and structure of the N-terminal domain of *Escherichia coli* initiation factor 3. Eur. J. Biochem..

[31] Pon C., Cannistraro S., Giovane A., Gualerzi C.O. (1982). Structure-function relationship in *Escherichia coli* initiation factors. Environment of the Cys residue and evidence for a hydrophobic region in initiation factor IF3 by fluorescence and ESR spectroscopy. Arch. Biochem. Biophys..

[32] Pon C.L., Gualerzi C.O. (1974). Effect of initiation factor 3 binding on the 30S ribosomal subunits of *Escherichia coli*. Proc. Natl. Acad. Sci. USA.

[33] Milon P., Maracci C., Filonava L., Gualerzi C.O., Rodnina M.V. (2012). Real-time assembly landscape of bacterial 30S translation initiation complex. Nat. Struct. Mol. Biol..

[34] Milon P., Carotti M., Konevega A.L., Wintermeyer W., Rodnina M.V., Gualerzi C.O. (2010). The ribosome-bound initiation factor 2 recruits initiator tRNA to the 30S initiation complex. EMBO Rep..

[35] Tsai A., Petrov A., Marshall R.A., Korlach J., Uemura S., Puglisi J.D. (2012). Heterogeneous pathways and timing of factor departure during translation initiation. Nature.

[36] Qin D., Fredrick K. (2009). Control of translation initiation involves a factor-induced rearrangement of helix 44 of 16S ribosomal RNA. Mol. Microbiol..

[37] Elvekrog M.M., Gonzalez R.L. (2013). Conformational selection of translation initiation factor 3 signals proper substrate selection. Nat. Struct. Mol. Biol..

[38] Milon P., Konevega A.L., Peske F., Fabbretti A., Gualerzi C.O., Rodnina M.V. (2007). Transient kinetics, fluorescence, and FRET in studies of initiation of translation in bacteria. Methods Enzymol..

[39] Wilson D.N. (2009). The A-Z of bacterial translation inhibitors. Crit. Rev. Biochem. Mol. Biol..

[40] Davies J., GILBERT W., Gorini L. (1964). Streptomycin, suppression, and the code. Proc. Natl. Acad. Sci. USA.

[41] Gorini L., Gundersen W., Burger M. (1961). Genetics of regulation of enzyme synthesis in the arginine biosynthetic pathway of *Escherichia coli*. Cold Spring Harb. Symp. Quant. Biol..

[42] Tsai A., Uemura S., Johansson M., Puglisi E.V., Marshall R.A., Aitken C.E., Korlach J., Ehrenberg M., Puglisi J.D. (2013). The impact of aminoglycosides on the dynamics of translation elongation. Cell Rep..

[43] Gromadski K.B., Rodnina M.V. (2004). Streptomycin interferes with conformational coupling between codon recognition and GTPase activation on the ribosome. Nat. Struct. Mol. Biol..

[44] Luzzatto L., Apirion D., Schlessinger D. (1968). Mechanism of action of streptomycin in *E. coli*: Interruption of the ribosome cycle at the initiation of protein synthesis. Proc. Natl. Acad. Sci. USA.

[45] La Teana A., Gualerzi C.O., Brimacombe R. (1995). From stand-by to decoding site. Adjustment of the mRNA on the 30S ribosomal subunit under the influence of the initiation factors. RNA.

[46] La Teana A., Pon C.L., Gualerzi C.O. (1993). Translation of mRNAs with degenerate initiation triplet AUU displays high initiation factor 2 dependence and is subject to initiation factor 3 repression. Proc. Natl. Acad. Sci. USA.

[47] Grigoriadou C., Marzi S., Pan D., Gualerzi C.O., Cooperman B.S. (2007). The translational fidelity function of IF3 during transition from the 30 S initiation complex to the 70 S initiation complex. J. Mol. Biol..

[48] Moazed D., Noller H.F. (1987). Interaction of antibiotics with functional sites in 16S ribosomal RNA. Nature.

[49] Fabbretti A., Schedlbauer A., Brandi L., Kaminishi T., Giuliodori A.M., Garofalo R., Ochoa-Lizarralde B., Takemoto C., Yokoyama S., Connell S.R. (2016). Inhibition of translation initiation complex formation by GE81112 unravels a 16S rRNA structural switch involved in P-site decoding. Proc. Natl. Acad. Sci. USA.

[50] Fabbretti A., Gualerzi C.O., Brandi L. (2011). How to cope with the quest for new antibiotics. FEBS Lett..

[51] Brandi L., Fabbretti A., Milon P., Carotti M., Pon C.L., Gualerzi C.O. (2007). Methods for identifying compounds that specifically target translation. Methods Enzymol..

[52] Fabbretti A., He C.-G., Gaspari E., Maffioli S., Brandi L., Spurio R., Sosio M., Jabes D., Donadio S. (2015). A derivative of the thiopeptide GE2270A highly selective against *Propionibacterium acnes*. Antimicrob. Agents Chemother..

[53] Brandi L., Maffioli S., Donadio S., Quaglia F., Sette M., Milon P., Gualerzi C.O., Fabbretti A. (2012). Structural and functional characterization of the bacterial translocation inhibitor GE82832. FEBS Lett..

[54] Fabbretti A., Brandi L., Petrelli D., Pon C.L., Castanedo N.R., Medina R., Gualerzi C.O. (2012). The antibiotic Furvina(R) targets the P-site of 30S ribosomal subunits and inhibits translation initiation displaying start codon bias. Nucleic Acids Res..

[55] Kaminishi T., Schedlbauer A., Fabbretti A., Brandi L., Ochoa-Lizarralde B., He C.-G., Milon P., Connell S.R., Gualerzi C.O., Fucini P. (2015). Crystallographic characterization of the ribosomal binding site and molecular mechanism of action of Hygromycin A. Nucleic Acids Res..

[56] Guex N., Peitsch M.C. (1997). SWISS-MODEL and the Swiss-PdbViewer: An environment for comparative protein modeling. Electrophoresis.

[57] Pettersen E.F., Goddard T.D., Huang C.C., Couch G.S., Greenblatt D.M., Meng E.C., Ferrin T.E. (2004). UCSF Chimera—A visualization system for exploratory research and analysis. J. Comput. Chem..

[58] Ban N., Beckmann R., Cate J.H.D., Dinman J.D., Dragon F., Ellis S.R., Lafontaine D.L.J., Lindahl L., Liljas A., Lipton J.M. (2014). A new system for naming ribosomal proteins. Curr. Opin. Struct. Biol..

