# Supplementary material for: Conformational Response of 30S-bound IF3 to A-Site Binders Streptomycin and Kanamycin
Source: Antibiotics (Basel). 2016 Dec 13;5(4):38. doi: 10.3390/antibiotics5040038 (PMC5187519; doi:10.3390/antibiotics5040038)
Supplement: Supplementary file 1 [file antibiotics-05-00038-s001.pdf]

Supplementary Materials: Conformational Response of 30S-bound IF3 to A-Site Binders Streptomycin and Kanamycin

Roberto Chulluncuy, Carlos Espiche, Jose Alberto Nakamoto, Attilio Fabbretti and Pohl Milón

Table S1. Distances from h44 to h45 of residues in the engaged state, disengaged state, and IF1-bound structures.

16S rRNA Residues		Distance (Å) [1]		
**h44**	**h45**	**Engaged [2]**	**Disengaged [3]**	**IF1-Bound [4]**
G1497 O2′	A1519 N1	4.0	5.8	4.0
G1497 O2′	A1518 N1	2.7	5.2	2.7
G1496 O2′	G1517 N1	3.2	9.0	3.2

[1] Distance measured with chimera [1]; [2] 30S with Paromomycin, PDB: 1FJG [2]; [3] 30S with streptomycin, PDB: 4DR3 [3]; [4] 30S with IF1, PDB: 1HR0 [4].

Table S2. Summary of structural counter effects between streptomycin and IF1 on the 30S subunit.

30S Subunit	Variable	Streptomycin [3]	IF1 [4]
h44	A1492-A1493	Unaffected	Flipped-out (towards A-site)
	A1414-G1487	Stabilized	Destabilized
	U1413-G1486	Stabilized	Destabilized
h45	G1517-C1496	Disengaged	Engaged
Platform	30S–IF3DL	Open	Close
	IF3 dissociation [1]	Rapid	Slow
	Subunit joining [1]	Rapid	Slow

[1] Measured on 30S IC formed with non-canonical mRNAs [5].

Table S3. Structures used for representations. All structures were obtained from the Protein Data Bank [6].

PDB	Molecules Present	Method	Resolution (Å)	Reference
1HR0	30S–IF1	X-ray diffraction	3.2	[4]
1TIF	IF3 NTD	X-ray diffraction	1.8	[7]
2IFE	IF3 CTD	NMR		[8]
4DR1	apo30S Subunit	X-ray diffraction	3.6	[3]
4DR3	30S–Streptomycin	X-ray diffraction	3.35	[3]
2ESI	A-site fragment–Kanamycin	X-ray diffraction	3	[9]
1FJG	30S–Streptomycin–Paromomycin–Spectinomycin	X-ray diffraction	3	[2]

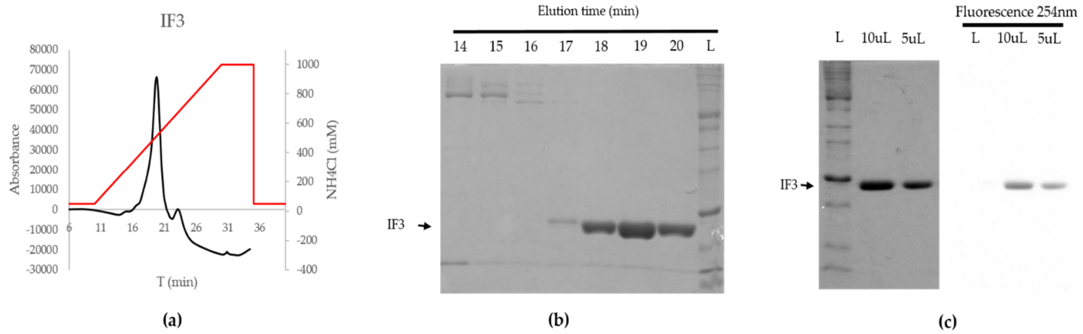

Figure S1. Purification and fluorescence labeling of IF3$_{E166C}$. (**a**) IF3$_{E166C}$ absorbance (290 nm) chromatogram (black) and NH$_4$CL gradient (red) used for the cation exchange chromatograph. Supernatants were manually loaded to the column (1 mL column volume) and subsequently subjected to a linear NH$_4$Cl gradient (0.05–1 M) with a 1 mL/min flow in a Jasco HPLC system (Jasco, Tokyo, Japan). The gradient was prepared in Buffer A (50 mM Hepes pH 7.1, 10% Glycerol, 6 mM 2-Mercaptoethanol). (**b**) SDS-PAGE (15%) of collected fractions. Well numbers represent the retention time (min) of the sample and L, ladder. (**c**) SDS-PAGE of fluorescent-labeled IF3$_{E166C}$. Purity and efficiency of labeling was assayed by 15% SDS-PAGE, where fluorescence was observed under a UV trans-illuminator (right) and total protein by blue Coomassie staining (left), L: ladder (6–212 kDa, NEB, Ipswich, MA, USA).

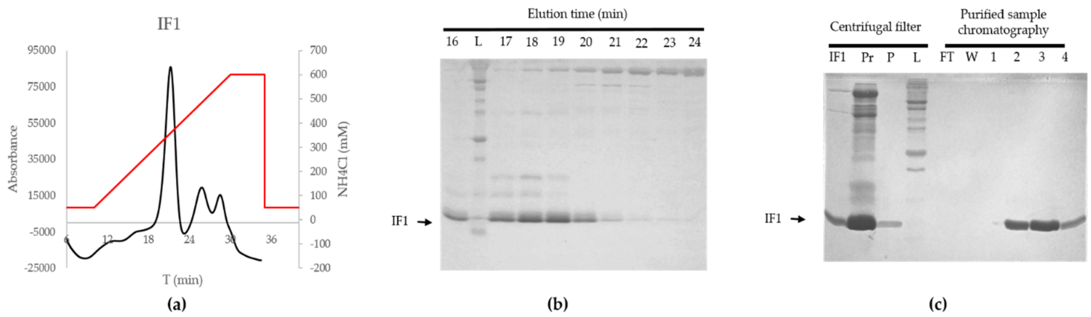

Figure S2. Purification of IF1. (**a**) IF1 absorbance chromatogram (black) and NH$_4$CL gradient (red) used for the cation exchange chromatograph. Supernatants were manually loaded to the column (1 mL column volume) and subsequently subjected to a linear NH$_4$Cl gradient (0.05–0.6 M) with a 1 mL/min flow in a Jasco HPLC system (Jasco, Tokyo, Japan). The gradient was prepared in Buffer A (50 mM Hepes pH 7.1, 10% glycerol, 6 mM 2-Mercaptoethanol). Protein elution was followed by absorbance at 290 nm. (**b**) SDS-PAGE 15% of collected fractions. Well numbers represent the retention time (min) of the fractions, and ladder (L). (**c**) IF1 clean-up and concentration using two complementary methods. In order to eliminate high molecular weight contaminants from the previous step, the pooled 15 mL preparation of IF1 was loaded to an Amicon® Ultra 15 mL centrifugal filter device with a nominal molecular weight limit (NMWL) of 30,000 Da following manufacturer indications. Notably, the Amicon filter followed by a step purification on the HiTrap SP HP column allowed the efficient elimination of high molecular weight contaminates and obtaining a concentrated IF1 preparation. IF1 *wt* showed >99% purity as judged by SDS-PAGE. Showed in the figure: a reference sample of IF1 (IF1), pre-filter IF1 (Pr), Post-filter IF1 (P), Ladder (L) (6–212 kDa, NEB) and the results of the concentration using Hi-trap column, flow through (FT), wash (W), elution fractions (1–4).

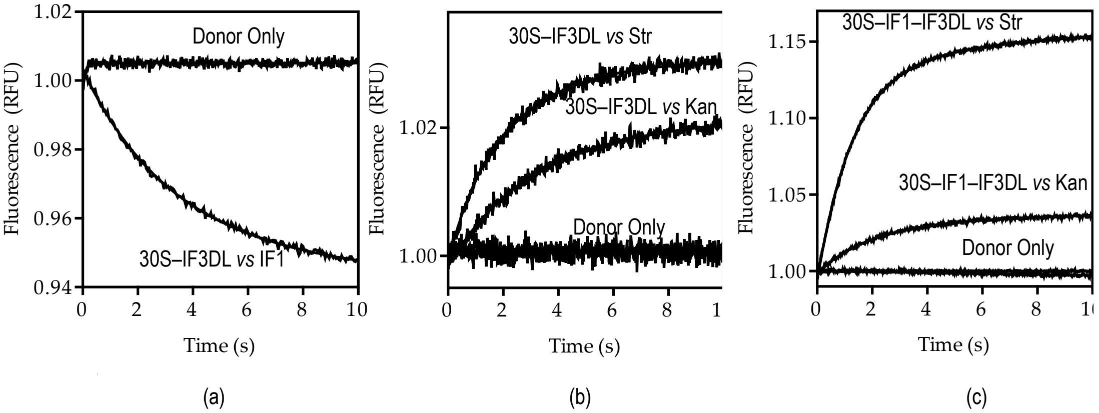

Figure S3. FRET controls for IF3DL sensing of A-site binders. (**a**) Time courses of IF1 (1 μM) binding to 0.1 μM 30S–IF3DL or 30S–IF3NAtto488 (donor only) complexes; (**b**) time courses of streptomycin and kanamycin binding to either 0.1 μM 30S–IF3DL or 30S–IF3NAtto488 (donor only) complexes; (**c**) same as (**b**) but in the presence of IF1. Seven to 10 independent traces were recorded and averaged. Continuous lines show best fits.

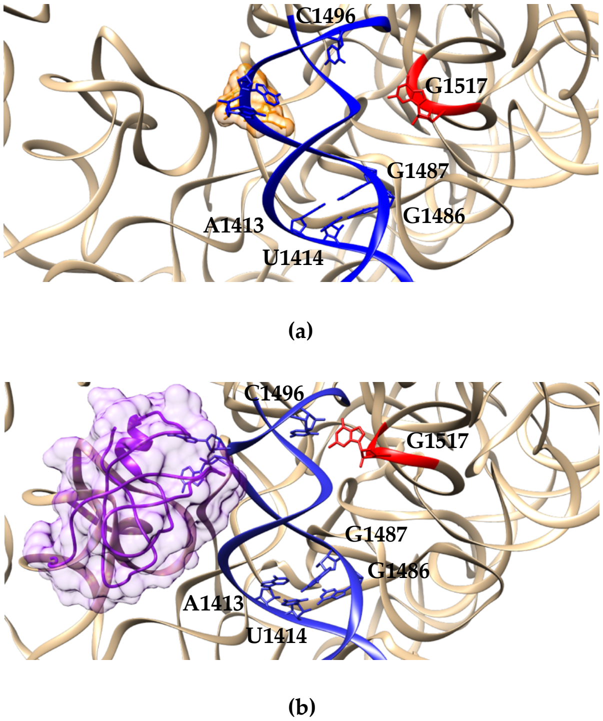

Figure S4. Possible structural changes induced by streptomycin and IF1 on the 30S subunit. (**a**) Streptomycin triggering a disengaged state between tetraloop of h45 (red) and h44 (blue) PDB (4DR3). Streptomycin is shown in orange. Changed nucleotides are shown. (**b**) IF1 inducing the engaged state between tetraloop of h45 and h44 (PDB 1HR0). Colors and residues are as in (**a**). IF1 is shown in purple.

References

1. Pettersen, E.F.; Goddard, T.D.; Huang, C.C.; Couch, G.S.; Greenblatt, D.M.; Meng, E.C.; Ferrin, T.E. UCSF Chimera—A visualization system for exploratory research and analysis. *J. Comput. Chem.* **2004**, *25*, 1605–1612.
2. Carter, A.P.; Clemons, W.M.; Brodersen, D.E.; Morgan-Warren, R.J.; Wimberly, B.T.; Ramakrishnan, V. Functional insights from the structure of the 30S ribosomal subunit and its interactions with antibiotics. *Nature* **2000**, *407*, 340–348.

3. Demirci, H.; Murphy, F.; Murphy, E.; Gregory, S.T.; Dahlberg, A.E.; Jogl, G. A structural basis for streptomycin-induced misreading of the genetic code. *Nat. Commun.* **2013**, *4*, doi:10.1038/ncomms2346.
4. Carter, A.P.; Clemons, W.M.; Brodersen, D.E.; Morgan-Warren, R.J.; Hartsch, T.; Wimberly, B.T.; Ramakrishnan, V. Crystal structure of an initiation factor bound to the 30S ribosomal subunit. *Science* **2001**, *291*, 498–501.
5. Milon, P.; Konevega, A.L.; Gualerzi, C.O.; Rodnina, M.V. Kinetic checkpoint at a late step in translation initiation. *Mol. Cell* **2008**, *30*, 712–720.
6. Berman, H.M.; Battistuz, T.; Bhat, T.N.; Bluhm, W.F.; Bourne, P.E.; Burkhardt, K.; Feng, Z.; Gilliland, G.L.; Iype, L.; Jain, S.; et al. The Protein Data Bank. *Acta Crystallogr. D Biol. Crystallogr.* **2002**, *58*, 899–907.
7. Biou, V.; Shu, F.; Ramakrishnan, V. X-ray crystallography shows that translational initiation factor IF3 consists of two compact alpha/beta domains linked by an alpha-helix. *EMBO J.* **1995**, *14*, 4056–4064.
8. Garcia, C.; Fortier, P.L.; Blanquet, S.; Lallemand, J.Y.; Dardel, F. Solution structure of the ribosome-binding domain of *E. coli* translation initiation factor IF3. Homology with the U1A protein of the eukaryotic spliceosome. *J. Mol. Biol.* **1995**, *254*, 247–259.
9. François, B.; Russell, R.J.M.; Murray, J.B.; Aboul-ela, F.; Masquida, B.; Vicens, Q.; Westhof, E. Crystal structures of complexes between aminoglycosides and decoding A site oligonucleotides: Role of the number of rings and positive charges in the specific binding leading to miscoding. *Nucleic Acids Res.* **2005**, *33*, 5677–5690.

© 2016 by the authors; licensee MDPI, Basel, Switzerland. This article is an open access article distributed under the terms and conditions of the Creative Commons by Attribution (CC-BY) license (http://creativecommons.org/licenses/by/4.0/).
